# Development of conductive fingermarks for forensic applications

**DOI:** 10.1007/s12024-024-00898-1

**Published:** 2024-10-24

**Authors:** Niamh E. Richards, Andrew Langley, Laura J. Vera Stimpson

**Affiliations:** 1 School of Law, Policing and Social Sciences, Canterbury Christ Church University, Kent, UK; 2 Division of Natural Sciences, University of Kent, Kent, UK

**Keywords:** Conductivity, Fingermarks, Forensic science

## Abstract

Fingermarks are an important form of evidence in forensic science, routinely used for identification or exclusion purposes within the criminal justice system. The increasing use of fingerprint recognition in technology and biosecurity, such as for unlocking devices and accessing banking information, highlights the need for forensic fingermark recovery methods that serve both traditional forensic needs and modern technological demands. Current fingerprint development techniques, however, are not designed to fulfil this dual-purpose. This paper presents a novel approach that introduces the use of conductive paint and silicone to develop, recover, and preserve latent, patent, and plastic fingermarks. The innovative method produces conductive casts that capture detailed ridge patterns, thereby facilitating forensic examination as well as being used for unlocking capacitive and ultrasonic fingerprint scanners in a range of mobile devices.

## Introduction

Biometrics are an integral facet of our everyday lives, being used in various societal applications and criminal investigations for identification purposes [1, 2]. Fingerprint recognition, as a form of biometric identification, has gained significant importance in technology and biosecurity. Biometric sensors, particularly those using fingerprint recognition systems, have gained widespread popularity in authenticating users in various daily applications including financial transactions, border security, and unlocking personal devices [3]. This popularity is attributed to the unique characteristics of fingerprints where the probability of finding two distinct fingers with identical ridge characteristics is exceptionally low [4]. However, this surge in use has prompted an increase in methods aimed at circumventing these systems, commonly referred to as ‘presentation attacks’ (ISO/IES 30107-1:2016) [5, 6].

Biometric systems are susceptible to different types of adversary attacks, including fingerprint obfuscation and impersonation. Instances of fingerprint obfuscation involve intentional alterations to the fingerprint pattern, achieved through methods such as cutting, abrading, burning ridge detail, or removing a portion of the skin from the fingertip [7]. Impersonation attacks can be achieved through several methods including, the use of cadaver fingers, 2D or 3D printed fingerprint targets, or gummy fingerprints (i.e. physical reproduction of an individual’s fingerprint characteristics) [8–10]. Among these, gummy fingerprints, also known as ‘spoofs’, are commonly employed and involve the use of various materials such as silicone, playdough, and gelatine for moulding and casting. The success rate of presentation attacks varies significantly for each material, influenced by factors such as the quality of ridge detail captured during the moulding process and damage to the casting materials over time [11, 12]. This damage ranges from deformation of the cast (e.g. playdough) to the limited shelf-life of casting materials such as gelatine, requiring careful handling and limited duration of use.

The application of fingermarks in forensic science has traditionally centred on their use for identification purposes, with more recent research exploring various aspects such as chemical analysis to extract information from the composition of fingermarks [13] and the enhancement of fingermark visualization through the use of nanoparticles [14–16]. However, research into using fingermarks for technological applications in forensic science, remains relatively unexplored. Notably, studies on these technological aspects are often not published in peer-reviewed academic sources, limiting the accessibility and rigor of the available literature. For instance, Cao (2016) [17] examined the potential for unauthorized access to mobile devices. The study involved creating fingerprint spoofs using latex, wood glue, and silver ink to replicate ridge patterns demonstrating their effectiveness in unlocking mobile devices.

Despite advancements in methods for improving fingermark development and the increasing knowledge of presentation attacks on biometric systems, a significant gap exists in exploring the broader forensic applications of these technologies. This gap may be partly attributed to challenges associated with the usability and stability of counterfeit fingerprints, as forensic protocols require the preservation of evidence without causing degradation. This paper introduces a novel approach for the non-destructive development, recovery, and preservation of fingermarks, facilitating their dual use as spoofs for capacitive and ultrasonic scanners as well as allowing ridge detail comparison for forensic examination purposes.

## Materials and methods

**Fig. 1:**
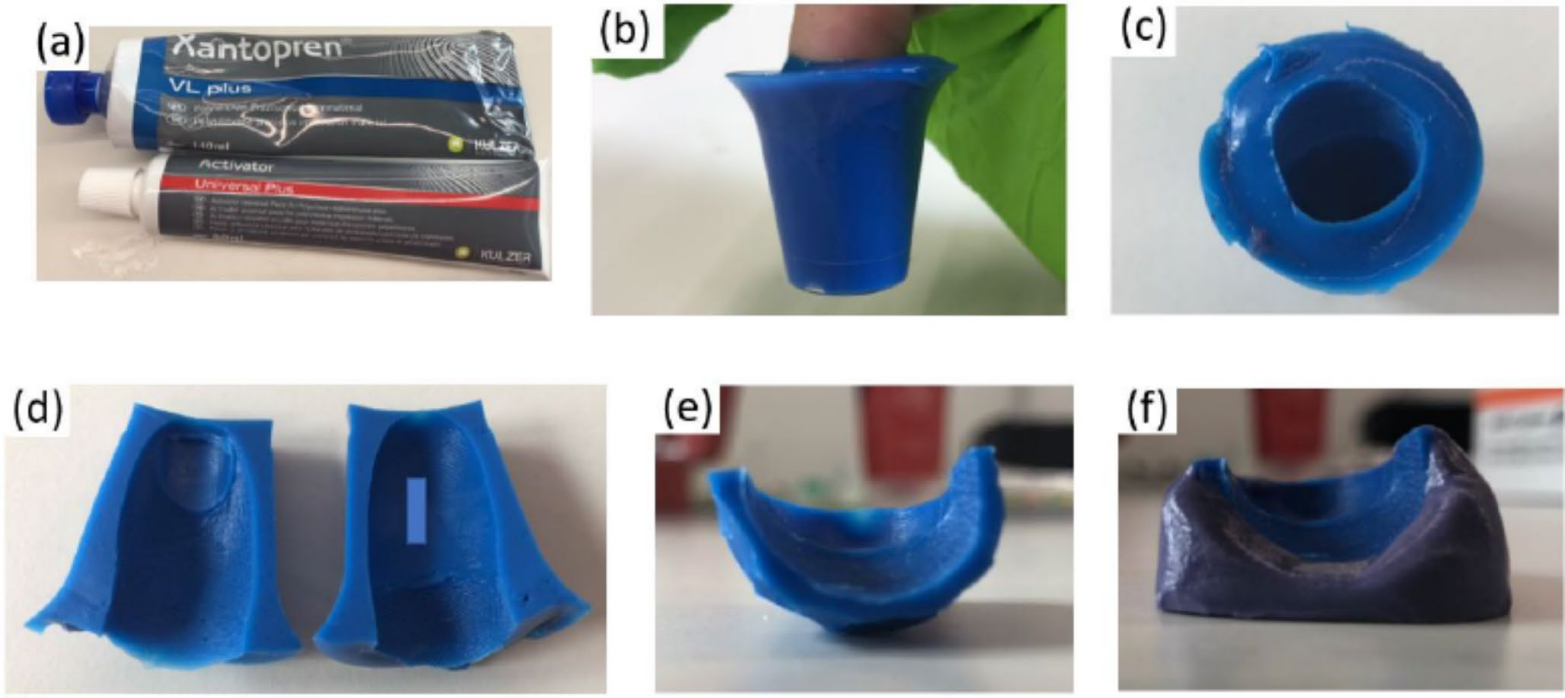
Ridge detail casting process showing **(a)** Xantopren^®^ VL plus, **(b)** placement of selected finger into the centre of the non-cured medium, **(c)** mould created after the removal of the finger from the cured medium, **(d)** incision along the midline of the cast, **(e)** volar surface of cast containing ridge detail and **(f)** cast is securely attached to an add-i-gum base

The casting of ridge detail was achieved using Xantopren^®^ VL Plus (Fig. 1 (a)), a silicone-based precision condensation curing impression material manufactured by KULZER. The preparation followed manufacturer instructions. The resulting mixture was transferred to a small disposable container.

The designated finger for casting was carefully inserted into the centre of the mixture (Fig. 1 (b)). During this process, the finger underwent gentle lateral movement to eliminate air bubbles. The finger was maintained in this position until the material solidified (approximately 5 min) (Fig. 1 (c)). After the setting process, a longitudinal incision was made along the midline of the cast, extending past the distal interphalangeal joint (DIPJ), leading to the separation of the dorsal and volar surface of the cast (Fig. 1 (d)). The cast of the dorsal surface was discarded, while the cast of the volar surface (Fig. 1 (e)) was placed onto an add-i-gum putty base (Fig. 1 (f)) to improve handling.

Conductive casts were produced using a composite comprising a high-resolution dental vinylpolysiloxane silicone (Provil^®^ novo light regular set, manufactured by KULZER) and conductive paint containing a water-based dispersion of carbon pigment in natural resin, manufactured by Bare Conductive. The preparation involved thorough mixing of Provil^®^, activator and conductive paint in a ratio of 2:1:1, followed by application onto the Xantopren casts. After application, the conductive casts remained undisturbed until the medium had undergone complete curing (approximately 20 min), at which point they were extracted through a peeling process.

Testing was conducted using a range of mobile devices, encompassing both Android and iOS platforms. These devices were equipped with diverse fingerprint recognition technologies, namely optical scanners (Oppo X3, and Samsung A71), ultrasonic scanners (Samsung S10, S21, S22 and S24 Ultra), and capacitive scanners (iPhone 6, 7, and 8). The study involved a diverse group of participants’, encompassing different genders and age ranges. Testing was performed both on controlled devices and participants’ personal devices.

A registered fingerprint identification expert conducted a detailed comparison of the friction ridge characteristics. The examination was performed using a linen tester with 8x magnification to enhance the visibility of the ridge details.

This study was approved by the local ethics committee. To ensure participant anonymity and comply with ethical guidelines, all fingermark images have been partly obscured.

## Results and discussion

### Casting

A three-dimensional (3D) cast was used to capture intricate friction ridge patterns and achieve a precise replication of the finger’s anatomical shape. Xantopren, commonly used in forensic science for recovering microdetail from toolmark impressions, was selected due to its known capabilities of capturing detail down to 1 μm [18]. This choice was fitting considering the typical thickness of fingermark ridges range from 100 to 300 μm [19].

Casts produced consistently captured a high level of ridge detail. Additionally, the use of Xantopren in its initial liquid state facilitated thorough coverage of the finger, thereby minimizing the formation of air bubbles.

### Conductive casts and quality of fingermark development

To ensure comprehensive coverage of all ridges within the cast, a medium was needed to facilitate the dispersion of conductive paint during application. For this purpose, Provil^®^ was doped with conductive paint. Successful doping required an adjustment to the manufacturer’s guidance for the ratio of Provil^®^ to activator. Specifically, a ratio of 2:1:1 for Provil^®^, activator and conductive paint was optimal for facilitating compound hardening, ensuring thorough dispersion of conductive paint throughout the cast.

Following mixing, the doped Provil^®^ was placed into a disposable syringe and applied to the cast using a side-to-side motion ensuring a slight overlap during the application process. Curing times for the doped Provil^®^ were longer compared to the undoped compound (< 60 min).

**Fig. 2:**
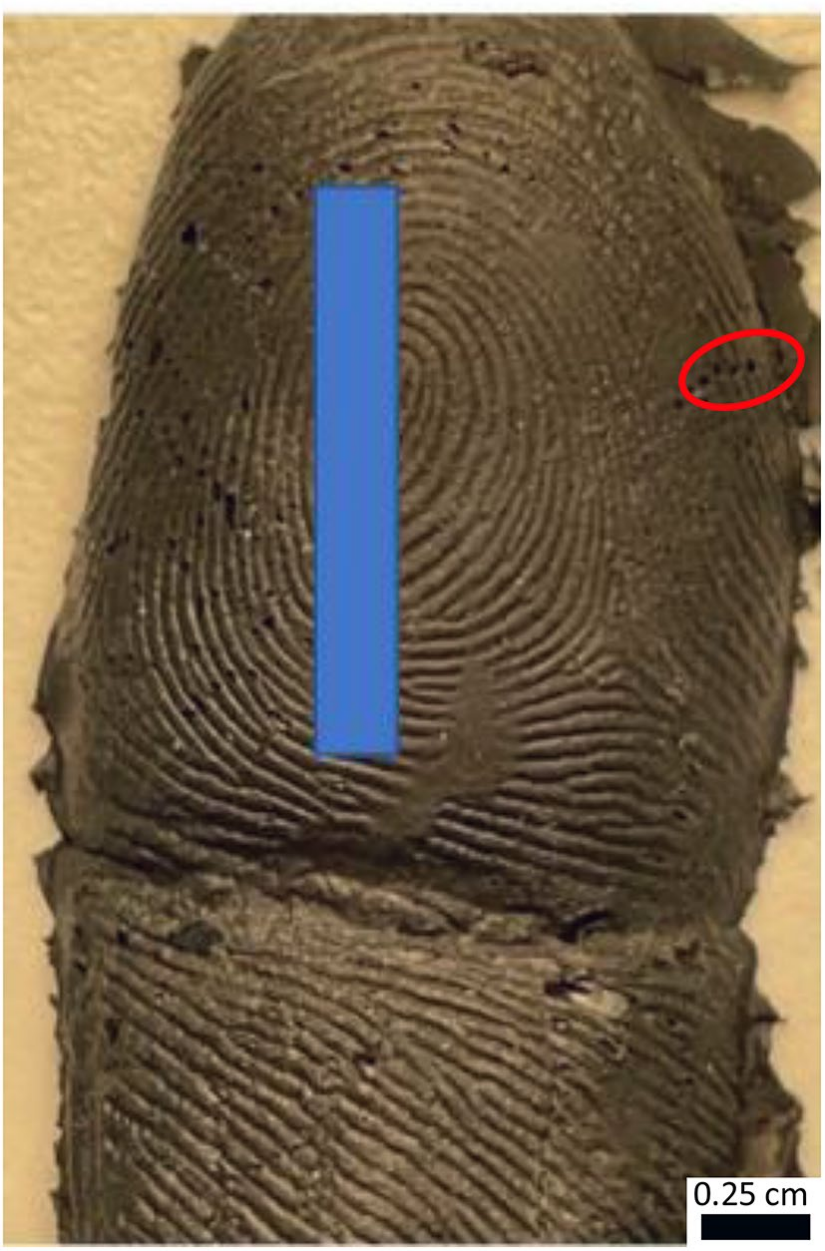
Conductive cast created using Provil^®^ doped with conductive paint. Red circle highlights void areas created in the cast as a result of air pockets

The conductive cast produced an accurate reproduction of friction ridges (Fig. 2). Despite minor instances of incomplete or absent detail in some areas, the overall quality of the reproduced ridges remained high. Consequently, these casts possess the potential to be utilized for identification purposes with minimal difficulty.

However, the three-dimensional characteristics of the cast posed challenges during photographic documentation, particularly in capturing intricate details of the fingermark. These difficulties were exacerbated by factors such as colour and shininess of the cast, which further impeded visualization methods.

**Fig. 3:**
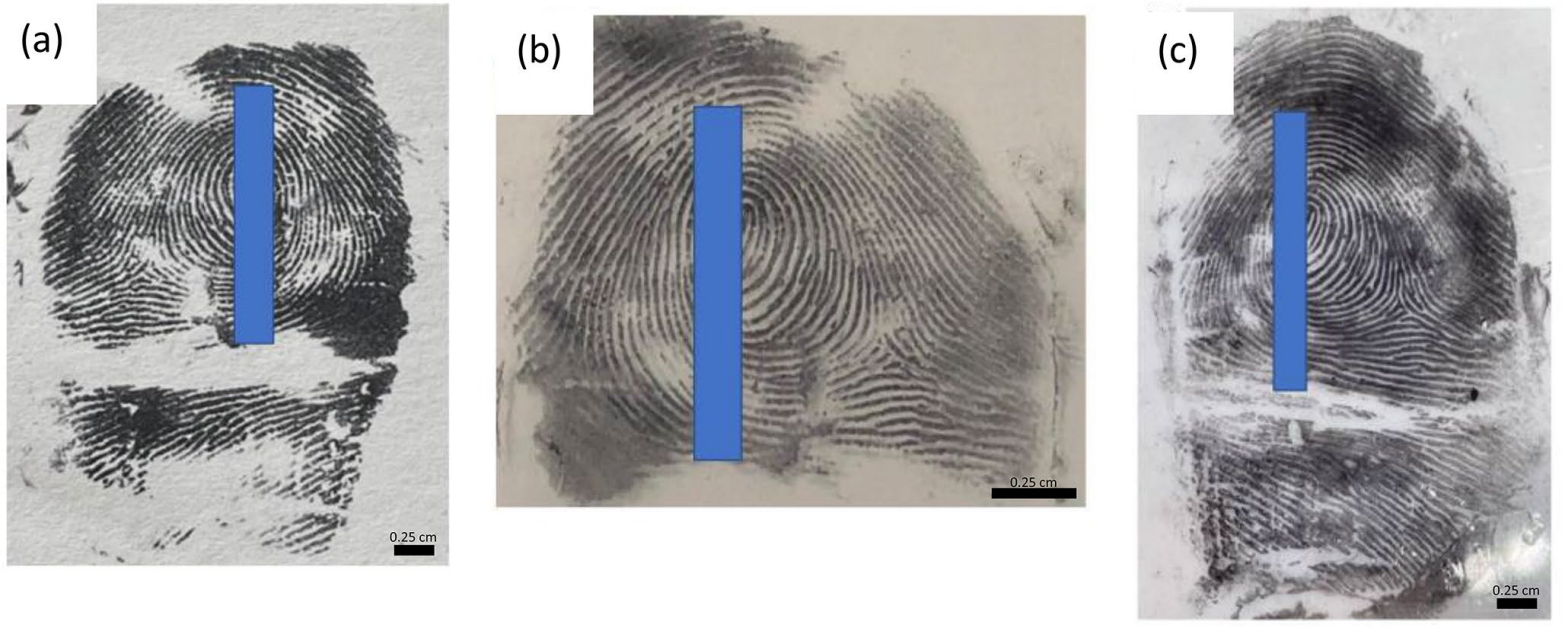
Images showing the results of using a conductive cast to create impressions in **(a)** black ink; **(b)** impression after application of magnetic powder and performing a single lift; **(c)** overlay of two magnetic powder lifts

To improve visualization, the conductive cast was inked using the same technique employed in capturing suspect fingerprints, which entailed coating the conductive cast with black ink and delicately rolling it from side to side. Partial transfer of ridge detail was achieved (Fig. 3 (a)), although void areas were evident. While the conductive casts displayed a certain level of flexibility, it did not match that of human fingertips. Consequently, the presence of voids can be attributed to the reduced flexibility, which prevented the cast from achieving full contact with the surface of the paper.

To overcome this, a small amount of magnetic powder was applied to the conductive cast using a magnetic wand. The conductive cast was then rolled over a piece of JLar tape and affixed onto a transparent acetate sheet using a roller. Consistent with earlier observations, some voids were encountered (Fig. 3 (b)). The use of transparent materials (acetate and JLar) enabled the recovery of multiple lifts, facilitating the overlay of retrieved fingermarks (Fig. 3 (c)) for comparative analysis. This showed clear third level detail, where the fingermark would be easily identifiable.

### Testing of conductive casts

**Fig. 4:**
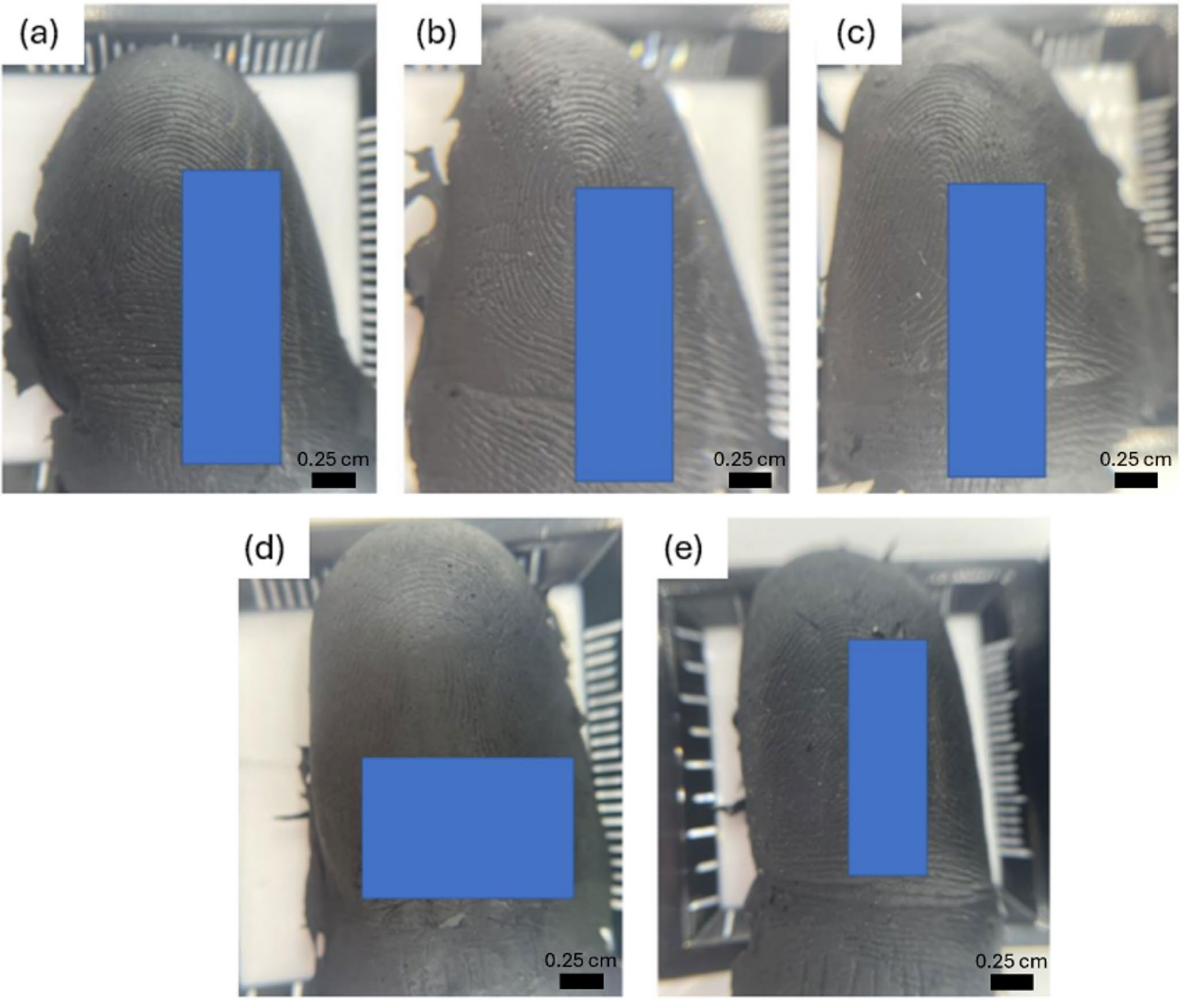
Conductive casts created from participants showing **(a - c)** good friction ridge projection and **(d – e)** poor friction ridge projection

The conductive casts were tested using a range of devices, yielding varying results (Table 1). Notably, all devices equipped with ultrasonic scanners were successfully unlocked. Conversely, a mixed outcome was observed with capacitive scanners, and all attempts to unlock optical scanners using the conductive casts proved unsuccessful. With optical scanners, although the conductive cast was successfully detected by the sensor, indicating sufficient conductivity for sensor recognition, it did not enable successful unlocking. The mixed outcomes observed with capacitive scanners can be attributed to two primary factors. Firstly, the integration of the fingerprint sensor into the home button of the device caused a slight recess. This recess presented challenges when positioning the conductive cast to ensure close contact with the sensor. Secondly, unsuccessful attempts with capacitive scanners were associated with casts exhibiting poor friction ridge projection (Fig. 4 (d – e)). The projection of friction ridges is influenced by various factors including age, handwashing habits, and occupational activities [20]. Furthermore, a decrease in collagen production as individuals age results in a reduction of friction ridge projection [20, 21].

**Table 1:** Summary of devices tested

Mobile device	Operating system	Fingerprint sensor type	Unlocking
iPhone 8 plus	iOS	Capacitive	Yes
iPhone 7	iOS	Capacitive	Yes
iPhone 6	iOS	Capacitive	Yes
iPhone SE	iOS	Capacitive	No
iPad Air 2	iOS	Capacitive	Variable
Samsung Galaxy S24 ultra	Android	Ultrasonic	Yes
Samsung Galaxy S22 ultra	Android	Ultrasonic	Yes
Samsung Galaxy S21 ultra	Android	Ultrasonic	Yes
Samsung Galaxy S7	Android	Capacitive	Yes
Samsung A71	Android	Optical	No
OPPO X3 lite	Android	Optical	No
PC scanner	Windows		Yes

### Applicability to exhibits

In forensic practice, fingermarks are typically categorized into three primary types: latent, patent, and plastic. While latent and patent fingermarks are characterized by a two-dimensional nature, plastic fingermarks exhibit a three-dimensional structure. To evaluate the efficacy of the conductive fingermark method for the recovery and preservation of fingermarks, a series of exhibits commonly encountered in everyday life were investigated. Testing of fingermarks was conducted using the same donor on both capacitive (iPhone 8 plus) and ultrasonic (Samsung S24 Ultra) fingerprint scanners.

#### Plasticine

**Fig. 5:**
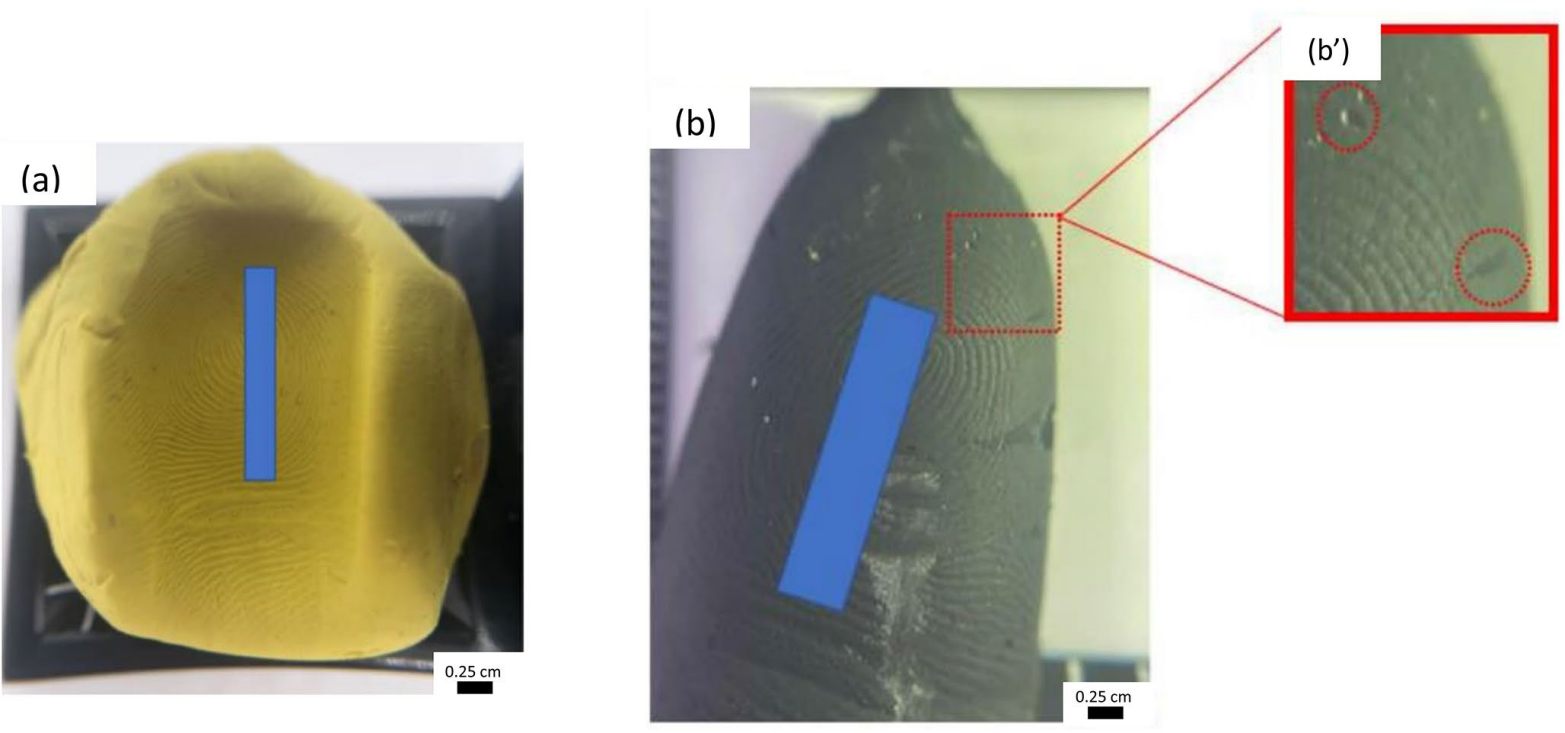
Image showing **(a)** fingermark deposited in plasticine and **(b)** conductive cast recovered from plasticine where voids present in the conductive cast (b’)

The plasticine was manually kneaded for approximately two minutes until it reached body temperature, after which it was shaped into a ball. A fingermark was deposited onto the surface, resulting in the capture of ridge detail (Fig. 5 (a)). Following this, the conductive casting procedure was applied, allowing for the capture of minutiae characteristics; however, these were insufficient to provide a coincident sequence. This limitation can be attributed to the contrast and colouration of the conductive cast (Fig. 5 (b)). Minor voids were observed due to surface imperfections on the plasticine which transferred to the conductive cast ((Fig. 5 (b’)). Despite these imperfections, the conductive cast effectively facilitated the unlocking of both capacitive and ultrasonic scanners tested.

#### Modelling dough

**Fig. 6:**
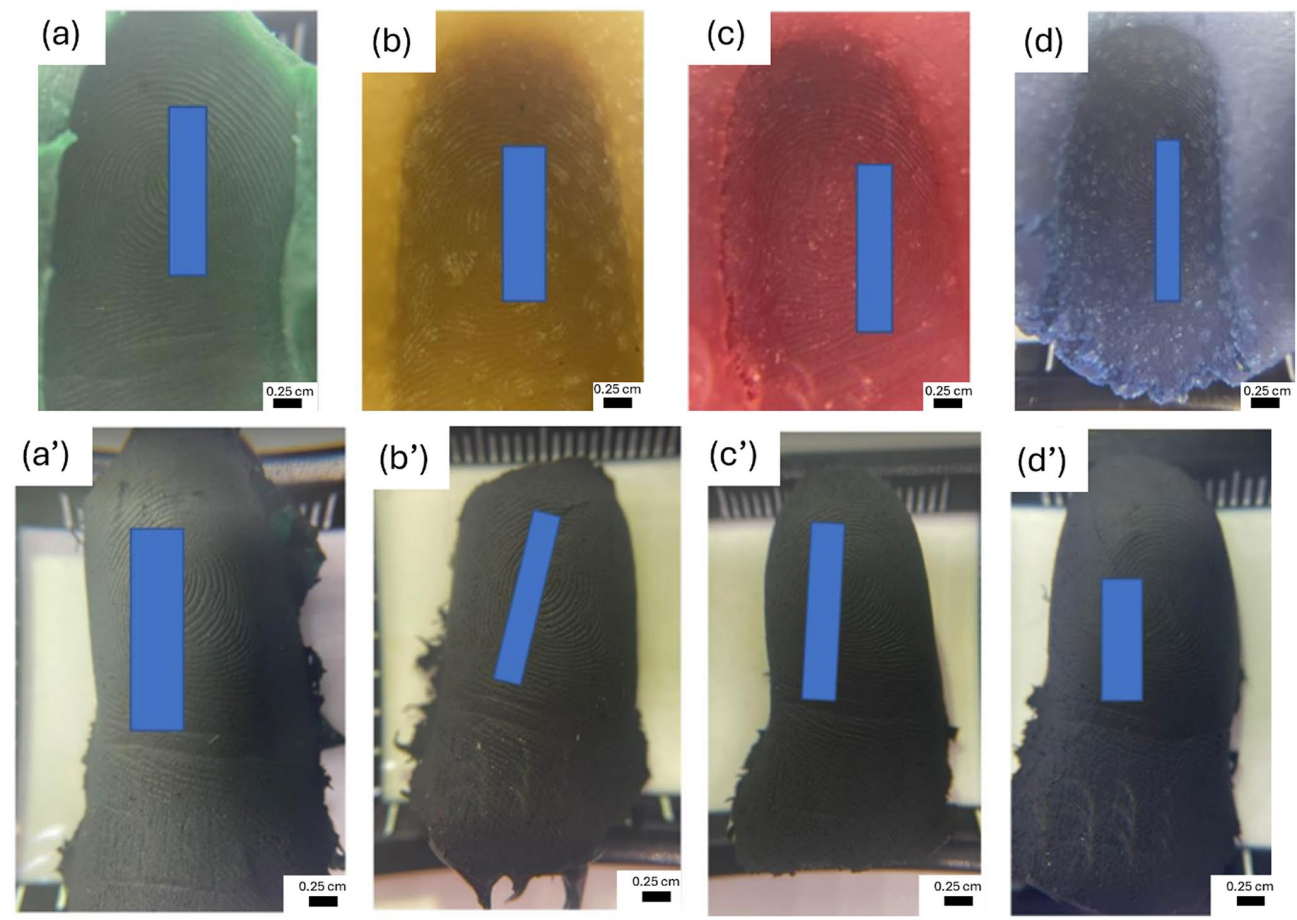
Fingermarks deposited onto modelling dough when **(a)** fresh, **(b)** dried after deposition (24 h), **(c)** left 30 min before deposition and **(d)** left 3 h before deposition. Images **(a’ – d’)** show conductive casts recovered from modelling dough when **(a’)** fresh, **(b’)** dried after deposition (24 h), **(c’)** left 30 min before deposition and **(d’)** left 3 h before deposition

The modelling dough exhibited greater malleability compared to plasticine, requiring minimal kneading. The material was shaped into a small ball prior to fingermark application. The properties of modelling dough changed upon exposure to air; therefore observations were made under various conditions: (1) fingermarks deposited immediately after kneading (Fig. 6 (a)), (2) fingermark deposited immediately after kneading and left for 24 h under ambient conditions (Fig. 6 (b)), (3) fingermarks deposited after the material was left under ambient conditions for 30 min (Fig. 6 (c)) and (4) fingermarks deposited after the material was left under ambient conditions for 3 h (Fig. 6 (d)). High-quality fingermarks were obtained when the impressions were made promptly after kneading. However, a decline in impression quality was observed (Fig.’6 (b - d)) as environmental exposure time increased. This is attributed to the loss of moisture which caused shrinkage and decreased the malleability of the material. This loss in moisture coincided with the appearance of dots on the surface of the modelling dough.

After the fingermarks were deposited and allowed to age, the conductive cast recovery technique was applied. The results (Fig. 6a’–b’) showed that a good level of ridge detail was obtained from each exhibit. All conductive casts exhibited discernible ridge flow patterns, with some minutiae characteristics visibly present.

However, the samples subjected to ageing under ambient conditions before fingermark deposition (Fig. 6 (c’-d’)) exhibited significantly less detail compared to the fresh samples. Subsequent testing showed that conductive casts obtained when the fingermark was applied immediately after kneading (Fig. 6 (a’-b’)) successfully unlocked the devices tested. Conversely, those in which the fingermark was applied after exposure to ambient conditions (Fig. 6 (c’-d’)) were unsuccessful in unlocking the devices tested.

#### Wax

Unscented white candle wax was melted and then allowed to cool. Once this began solidifying, indicated by a change in colour from clear to white, a fingermark was deposited onto the wax surface. The wax was then allowed to fully solidify.

**Fig. 7:**
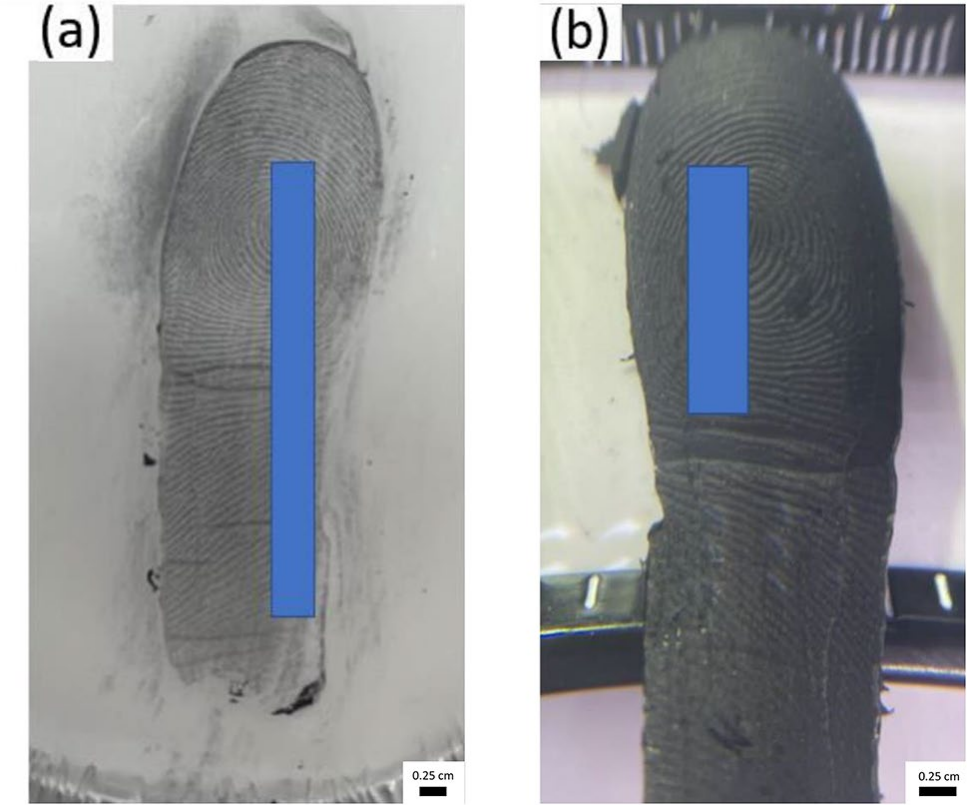
Image showing **(a)** fingermark deposition in wax where ridge detail has been enhanced using black magnetic powder and **(b)** conductive cast recovered from wax

The wax captured an excellent level of ridge detail, as shown in Fig. 7 (a). The conductive cast recovery technique was applied to this exhibit which resulted in casts retaining a comparable level of ridge detail Fig. 7 (b). Conductive casts obtained from wax exhibited excellent clarity and minutiae details, facilitating the establishment of a coincident sequence. The conductive cast obtained effectively facilitated the unlocking of both capacitive and ultrasonic scanners tested.

#### Anti-climb paint

**Fig. 8:**
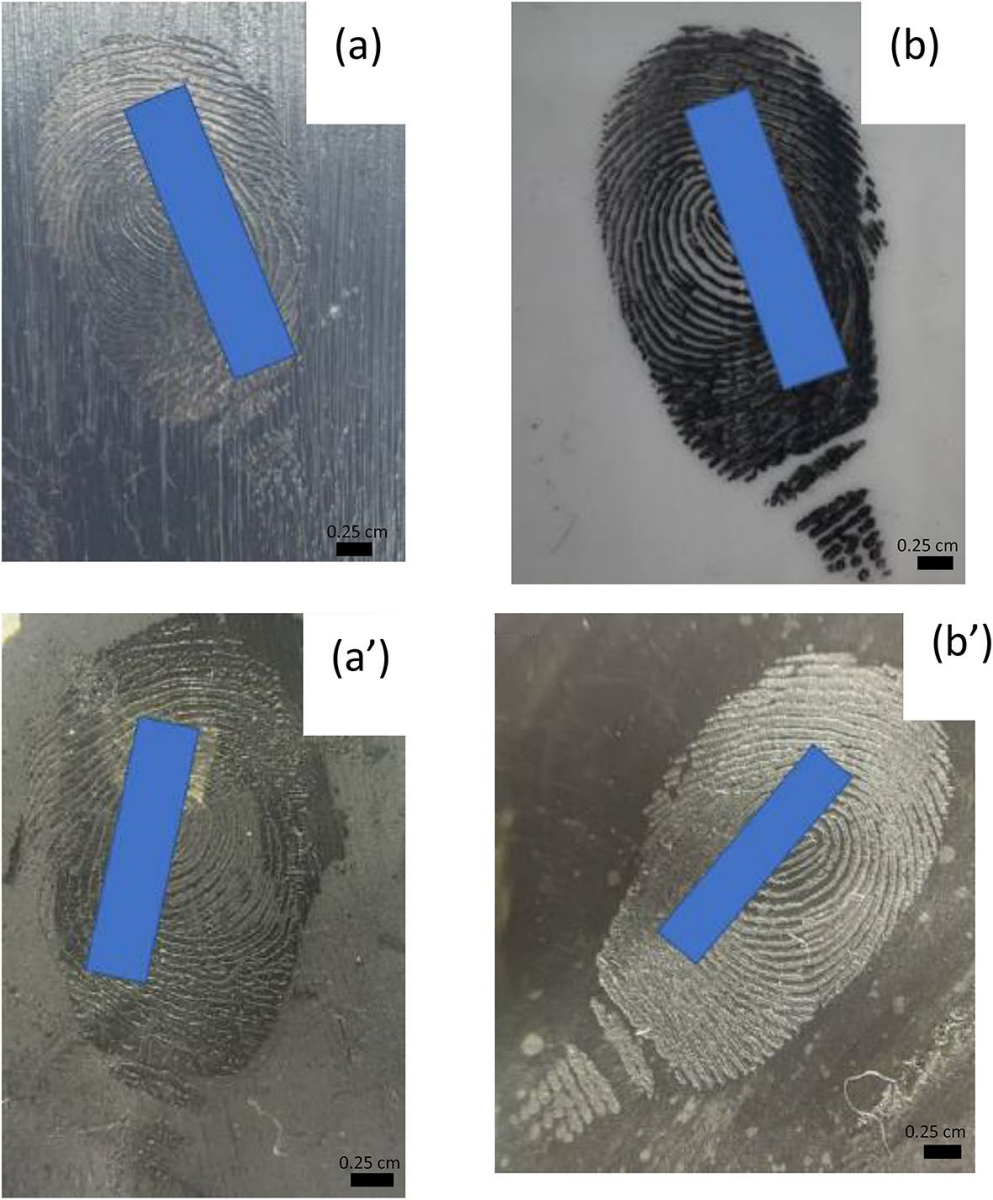
Fingermarks deposited in anti-climb paint and developed using cyanoacrylate ester fuming (CEF). Image **(a)** shows the developed fingermark from primary transfer, and image **(b)** from secondary transfer. Images (a’) and (b’) show the conductive casts recovered from CEF for the primary and secondary transfers, respectively

Anti-climb paint, a petroleum gel-based substance, is formulated to retain its wet and slippery properties indefinitely upon application to a surface [22]. However, tackiness reduces over time [22], therefore the anti-climb paint was applied to an acetate sheet and left for several hours, prior to fingermark deposition, after which primary and secondary fingermarks were deposited. Due to the non-drying nature of anti-climb paint, the recovery technique developed by Davatwal et al. [22] was employed (Fig. 8 (a-b)).

The primary transfer revealed a good level of detail (Fig. 8 (a)), with some third level characteristics clearly visible. However, a notably higher level of detail was obtained for the secondary transfer (Fig. 8 (b)). The conductive casting technique was applied to both primary and secondary transfers. The conductive casts (Fig. 8 (a’ – b’)) showed a good level of ridge detail, with the secondary transfer showing a higher level of detail.

When tested on capacitive and ultrasonic fingerprint scanners, the conductive cast obtained from the primary transfer failed to unlock the devices. In contrast, the conductive cast obtained from the secondary transfer successfully unlocked the devices tested. These results align with previous findings, highlighting the critical role of the quality of ridge detail in the success of this technique.

## Conclusion

Biometric systems, particularly those based on fingerprint recognition technology, have become an integral facet in society, facilitating fast authentication in various applications. Despite research efforts aimed at understanding and exploiting vulnerabilities within these systems for the development of spoofing techniques, their potential application within forensic science remains largely unexplored.

This study presents a novel methodology using a fingerprint casting technique, which allows for the capture of high-quality friction ridge detail. Additionally, the robustness of the material ensures prolonged usability with minimal concerns regarding storage and handling. The conductive properties of these casts enable their utilization as potential spoofing tools for ultrasonic and capacitive fingerprint scanners, while also allowing for these to be used for forensic fingerprint examination. Variations in success rates were observed, predominantly attributable to inherent differences in friction ridge projection, highlighting the importance of initial fingermark quality preceding the conductive casting process.

Furthermore, the study assessed the suitability of the conductive casting technique for various exhibit types encountered in forensic settings, including both 3D and 2D ridge detail. Similarly to previous findings, the quality of the deposited fingermark emerged as a decisive factor influencing the efficacy of device unlocking.
